# Salivary metabolic signatures of carotid atherosclerosis in patients with type 2 diabetes hospitalized for treatment

**DOI:** 10.3389/fmolb.2022.1074285

**Published:** 2022-12-21

**Authors:** Akito Sakanaka, Naoto Katakami, Masahiro Furuno, Hitoshi Nishizawa, Kazuo Omori, Naohiro Taya, Asuka Ishikawa, Shota Mayumi, Moe Inoue, Emiko Tanaka Isomura, Atsuo Amano, Iichiro Shimomura, Eiichiro Fukusaki, Masae Kuboniwa

**Affiliations:** ^1^ Department of Preventive Dentistry, Osaka University Graduate School of Dentistry, Suita, Japan; ^2^ Department of Metabolic Medicine, Osaka University Graduate School of Medicine, Suita, Japan; ^3^ Department of Biotechnology, Osaka University Graduate School of Engineering, Suita, Japan; ^4^ First Department of Oral and Maxillofacial Surgery, Osaka University Graduate School of Dentistry, Suita, Japan

**Keywords:** saliva, diabetes, atherosclerosis, carotid intima-media thickness (IMT), oxidative stress, metabolomics

## Abstract

Atherosclerosis is a life-threatening disease associated with morbidity and mortality in patients with type 2 diabetes (T2D). This study aimed to characterize a salivary signature of atherosclerosis based on evaluation of carotid intima-media thickness (IMT) to develop a non-invasive predictive tool for diagnosis and disease follow-up. Metabolites in saliva and plasma samples collected at admission and after treatment from 25 T2D patients hospitalized for 2 weeks to undergo medical treatment for diabetes were comprehensively profiled using metabolomic profiling with gas chromatography-mass spectrometry. Orthogonal partial least squares analysis, used to explore the relationships of IMT with clinical markers and plasma and salivary metabolites, showed that the top predictors for IMT included salivary allantoin and 1,5-anhydroglucitol (1,5-AG) at both the baseline examination at admission and after treatment. Furthermore, though treatment induced alterations in salivary levels of allantoin and 1,5-AG, it did not modify the association between IMT and these metabolites (*p*_interaction_ > 0.05), and models with these metabolites combined yielded satisfactory diagnostic accuracy for the high IMT group even after treatment (area under curve = 0.819). Collectively, this salivary metabolite combination may be useful for non-invasive identification of T2D patients with a higher atherosclerotic burden in clinical settings.

## Introduction

Atherosclerosis refers to the formation of fibrofatty lesions in artery walls, which can impede blood flow, leading to tissue ischemia. Atherosclerotic cardiovascular disease (CVD) is the leading cause of morbidity and mortality in patients with type 2 diabetes (T2D) (Mach et al., 2019). Furthermore, diabetes itself is an independent risk factor for CVD, and recent findings indicate that its presence increases that risk by approximately two-fold on average, though there are wide variations depending on the subject population and prophylactic treatment received (Sattar, 2013; Olesen et al., 2017). Therefore, long-term management of T2D patients requires early and accurate identification, as well as monitoring of those at high risk for CVD. Carotid ultrasonography is useful for evaluating subclinical atherosclerosis and various measurement results obtained with that method, including carotid intima-media thickness (IMT), have been shown to be predictive of CVD (Lorenz et al., 2007). However, it is not feasible to perform carotid ultrasonography for universal screening in patients with T2D, as specialized machines, skilled technicians, and patient restraint are required. Novel biomarkers that can better predict atherosclerosis burden and CVD risk are anticipated to facilitate early treatment induction, and reduce CVD-related morbidity and mortality associated with T2D.

Metabolites are small molecules known to reflect biological processes and their measurements are often utilized in clinical medicine as biomarkers for diagnosis, prognosis, and treatment efficacy (Wishart, 2019). Recent advancements in high-throughput technologies have allowed for systematic evaluation of a metabolome, a collection of metabolites, with regard to cardiometabolic changes, and several studies using blood metabolome have characterized disease-related metabolic pathways, including amino acid and fatty acid metabolism (Fan and Pedersen, 2021). In addition, state-of-the-art metabolomics data related to the risk of type 2 diabetes and its complications, analyzed from a wide variety of biological samples (plasma, serum, and urine), have been published (Sharma et al., 2013; Morze et al., 2022). Previously, we investigated the association of paired plasma and salivary metabolomic datasets from patients with T2D, and demonstrated the potential utility of salivary metabolites for evaluating systemic metabolic dysfunction (Sakanaka et al., 2021). However, there remains a lack of metabolomic studies that sought to address the complexity of T2D-CVD crosstalk, such as how atherosclerotic burden can alter metabolic profiles in plasma and saliva in T2D patients.

The aim of the present study was to identify multivariate covariation patterns between carotid atherosclerosis, and saliva and plasma metabolomes to develop non-invasive tools for prediction of atherosclerotic burden in T2D patients. To achieve this, comprehensive metabolomic profiling of plasma and saliva obtained from T2D patients was performed, and multivariate covariations with clinical markers of oral and systemic health were investigated. For the analyses, we employed a powerful multivariate method termed orthogonal partial least square (OPLS) to go beyond simple correlations. This method has been reported suitable for analysis of high-dimensional datasets where numerous variables are expected to be highly correlated (Wheelock and Wheelock, 2013). Using results obtained by modeling the association between IMT and clinical and metabolomic parameters with OPLS, this study presents a catalog of plasma and salivary metabolites that potentially reflect atherosclerotic burden. It is considered that the findings presented suggest the potential of salivary metabolites for evaluating cardiovascular risk in T2D patients.

## Materials and methods

### Study population

The present study was approved by the Osaka University Research Ethics Committee, and performed according to the principles of the Helsinki Declaration and STROBE guidelines for human observational studies. All participants gave written informed consent prior to enrollment and provided samples at Osaka University Medical Hospital. Participants with T2D, diagnosed using the criteria of the Japan Diabetes Society (Haneda et al., 2018), were recruited from November 2017 through March 2019 from among patients who visited the Department of Metabolic Medicine at Osaka University Medical Hospital for intensive diabetes treatment in an inpatient setting. Those with severe renal dysfunction or end-stage renal failure (serum creatinine >2.0 mg/dL), or under 50 years of age were excluded. All enrolled patients received comprehensive diabetes care, including intensive glycemic control, as well as blood pressure, dyslipidemia, and body weight control treatments while under hospitalization. Saliva, fasting blood, and urine samples were also obtained, and vital signs and weight were measured at admission (baseline) and again 2 weeks after treatment. Of 33 patients examined from November 2017 to March 2019, 25 met the criteria and were included in the study.

### Blood and urine sample collection, and laboratory measurements

Blood and urine samples were collected after an overnight fast at admission and again 2 weeks after treatment during hospitalization. Those were subjected to biochemical testing such as HbA1c and urine albumin, which were performed according to standard protocols. Furthermore, fasting plasma was collected and kept at 4°C in a freezer (CubeCooler^®^; Forte Grow Medical, Tochigi, Japan), and then frozen at −80°C, and used for metabolomics. All patients underwent anthropometric measurements and were asked to complete a variety of surveys regarding demographics, current and past medical history, medications, smoking history, and family history. Determinations of hypertension (defined as systolic blood pressure ≥130 mmHg, diastolic blood pressure ≥80 mmHg, or anti-hypertensive medication use), dyslipidemia [defined as serum low-density lipoprotein cholesterol (LDL-C) ≥120 mg/dL, serum triglycerides (TG) ≥150 mg/dL, high-density lipoprotein cholesterol (HDL-C) <40 mg/dL, or lipid-lowering medication use], and obesity (BMI ≥ 25 kg/m^2^) were based on the criteria of the Japan Diabetes Society.

### Carotid IMT measurement

Details of the carotid ultrasonic examination methods used have been presented (Omori et al., 2020). Briefly, a B-mode ultrasonography examination of the carotid artery was performed with a 7.5-MHz liner transducer. All scanning was conducted by experienced laboratory physicians using the same measuring method, in accordance with the guidelines of the Japan Society of Ultrasonics in Medicine (Terminology and Diagnostic Criteria Committee and Japan Society of Ultrasonics in Medicine, 2009). The thickest point for IMT in both common carotid arteries was separately determined, with the highest value defined as IMT for each individual in this study. This measurement was only performed at admission.

### Oral examination and saliva sample collection

All participants were asked to refrain from eating, drinking, or brushing their teeth for at least 1 h before undergoing the following procedures. Four calibrated licensed dentists performed oral examinations using techniques previously described (Sakanaka et al., 2021), and obtained saliva samples on the same day as blood and urine samples. For saliva output, each participant was asked to collect unstimulated whole saliva over a 10-min period in a 50-ml tube (Corning, NY, United States) kept on ice. Four diabetes patients with saliva output of less than 3 ml/10 min were asked to take 3 ml of distilled water (HPLC grade; Sigma-Aldrich, St. Louis, MO, United States) into their mouth and spit it out into a tube. By making the corrections described below regarding metabolomics analysis, we confirmed that exclusion of these four samples did not change the main results. After incubation on ice for 15 min, 1 and 0.1 ml of the aqueous layer were designated as the study sample and the quality control (QC) sample, respectively, which were then separately aliquoted into 2-ml tubes and maintained at 4°C in a CubeCooler^®^. They were subsequently frozen with liquid nitrogen and stored at −80°C until analysis.

### Saliva and plasma metabolomics

Metabolomics analysis was performed as previously described (Sakanaka et al., 2021). Briefly, saliva samples were thawed to 4°C, then vortexed and centrifuged (18,000 × *g*) for 3 min. Next, 0.8 ml of the aqueous layer was aliquoted and weighed, then 0.3 ml of that was transferred into a 2-ml glass vial (Nichiden-Rika Glass, Kobe, Japan) and kept at 4°C in a CubeCooler^®^. For extraction, 0.3 ml of a deaerated ribitol aqueous solution (0.02 mg/ml) was added as the internal standard. After incubation using an Eppendorf thermomixer (25°C, 1,000 rpm, 10 min), 1.4 ml of deaerated acetonitrile was added. After another incubation (25°C, 1,000 rpm, 10 min) and then centrifugation (4°C, 1800 × *g*) for 3 min, 1.6 ml of the supernatant was transferred to a 2-ml tube and dried in a vacuum concentrator (VC-96R; TAITEC, Koshigaya, Japan) for 30 min, then lyophilized overnight. For derivatization, a methoxyamine hydrochloride solution with pyridine was used at a concentration of 20 mg/ml, followed by silylation treatment with N-methyl-N-(trimethylsilyl)-trifluoroacetamide (MSTFA). Analysis using gas chromatography coupled with mass spectrometry (GC/MS) was performed using a GCMS-TQ8040 (Shimadzu, Kyoto, Japan) equipped with an AOC-20i autosampler (Shimadzu), a SKY™ liner (Restek, Bellefonte, PA, United States), and an InertCap 5MS/NP capillary column (0.25 mm × 30 m, 0.25 µm; GL Sciences, Tokyo, Japan), operated in full MS scan mode. QC samples consisting of an equimolar mixture of all saliva samples were injected every five samples to monitor MS signal drift and derivatization efficiency, followed by normalization with locally weighted scatter plot smoothing (LOWESS) in the subsequent data processing steps. GC-MS data were converted into ABF format, then processed using MS-DIAL (version 3.90) to perform feature detection, spectra deconvolution, metabolite identification, and peak alignment (Tsugawa et al., 2015). The acquired peak list was further normalized based on internal standard (ribitol) and sample weight (g/ml), as well as LOWESS algorithm. Metabolites from blanks and those with a coefficient of variation in QC samples above 30% were discarded. A total of 976 salivary metabolites were detected using this metabolomics platform, among which 142 were identified by matching retention time and fragmentation spectra to authentic standards. Plasma samples were prepared and analyzed with a GC-MS/MS-TQ8040 in multiple reaction monitoring mode, as previously described (Katakami et al., 2020; Taya et al., 2021). The obtained metabolomics data are available at Metabolomics Workbench (Study ID: ST001905 and ST001906).

### Statistical analysis

Using the SIMCA-P software package, v. 16 (Umetrics, Umeå, Sweden), an OPLS model was constructed with IMT as the Y response variable and all other parameters as X variables, which were all block-scaled by unit variance prior to analysis so that the influence of blocks of variables could be balanced in relation to their size. A seven-fold cross-validation was performed to avoid model over-fitting. Model quality and performance were assessed using *R* ^2^ (goodness of fit) and *Q* ^2^ (goodness of prediction) values, cross-validation analysis of variance (CV-ANOVA), and a permutation test (assessment of risk of over-fitting). The OPLS method can distinguish data variations correlated to the Y response variable from those orthogonal to Y response. Results thus obtained can assist with biological interpretation and enables establishment of a link between variations of variables and outcomes while removing information from other sources of variation. Variables reflecting the IMT variation were selected based on variable importance in projection of the predictive component (VIP predictive) of the OPLS model as well as Spearman’s correlation. The diagnostic ability of some parameters for IMT >1.6 mm was evaluated according to receiver operating characteristic (ROC) curve and area under the ROC curve (AUC) results. Cardiometabolic disease risk score was calculated as previously described (Wang et al., 2021a). Briefly, blood biomarker levels were first categorized into quintiles without distinction between pre- and post-treatment by ranking HbA1c, total cholesterol, TG, and high-sensitivity C-reactive protein (hsCRP) from lowest to highest with scores from 1 to 5. For HDL-C, the scoring was reversed. The cardiometabolic disease risk score was then calculated by summing those components, with a higher score indicating a higher risk of cardiometabolic disease. Differences as compared to the baseline regarding cardiometabolic disease risk score and salivary metabolite levels were analyzed with a paired *t* test. An interaction test was performed to determine whether treatment had an effect modification on the association between IMT and salivary metabolites. Spearman’s correlation was performed using the GraphPad Prism software package, v.8, and ROC curves and an interaction test were performed with the R package (v. 4.0.3).

## Results

**FIGURE 1:**
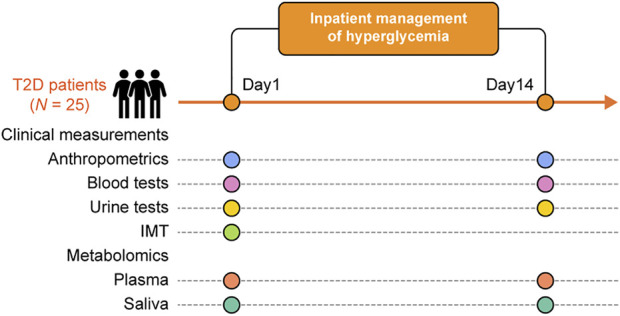
Overview of the experimental procedures. This study yielded clinical measures (IMT, anthropometrics, and blood and urine biochemical tests), and metabolome data from plasma and saliva at admission and after treatment for each of the 25 participants.

After applying the aforementioned exclusion criteria, 25 patients with T2D were available for statistical analysis (Figure 1) and their clinical characteristics are shown in Table 1. Using untargeted GC/MS, 142 salivary and 78 plasma metabolites were identified. Of those, when plasma indoleacetaldehyde and salivary indoleacetic acid were treated as the same due to structural similarity, 62 metabolites were found to be shared between them, seven of which showed a significant positive association at the baseline (Spearman’s correlation value > 0.3, *p* < 0.05). Among them, 1,5-anhydroglucitol (1,5-AG) demonstrated the strongest positive association between plasma and saliva (*r* = 0.76, *p* = 9.0 × 10^–5^). Overall, rich datasets comprised of three data blocks (44 clinical markers, 142 salivary, and 78 plasma metabolites) obtained at both the baseline and after treatment were generated.

**TABLE 1:** Main clinical characteristics of study participants.

Variables	Diabetes (*n* = 25)		
	Baseline	Week 2	*p*-value
Age, years	67.84 (3.75)	—	—
Females, *n* (%)	14 (56)	—	—
Diabetes duration, years	17.52 (11.76)	—	—
HbA1c, %	8.78 (1.84)	8.16 (1.44)	<0.001
Fasting plasma glucose, mg/dL	135.92 (41.07)	110.32 (19.47)	0.0075
Glycated albumin, %	23.36 (5.82)	19.67 (3.53)	<0.001
Obesity, *n* (%)	16 (64)	—	—
BMI, kg/m^2^	25.99 (3.12)	25.20 (3.01)	<0.001
Waist circumference, cm	97.30 (9.17)	—	—
Hypertension, *n* (%)	17 (68)	—	—
Systolic BP, mmHg	131.04 (18.10)	124.36 (12.31)	0.10
Diastolic BP, mmHg	74.48 (14.33)	74.92 (11.74)	0.87
Mean BP, mmHg	93.35 (13.90)	91.4 (10.90)	0.46
Dyslipidemia, *n* (%)	21 (84)	—	—
AST, U/L	27.8 (17.45)	24.04 (14.35)	0.0075
ALT, U/L	26.96 (16.24)	25.12 (19.34)	0.33
γ-GTP, U/L	40.64 (32.52)	32.96 (29.27)	<0.001
Triglycerides, mg/dL	137.76 (83.36)	96.56 (34.01)	0.0034
HDL cholesterol, mg/dL	54.28 (12.44)	52.24 (10.24)	0.11
LDL cholesterol, mg/dL	108.80 (30.02)	82.88 (23.45)	<0.001
Total cholesterol, mg/dL	187.96 (34.86)	154.68 (25.38)	<0.001
hs-CRP, mg/L	1676.32 (3109.72)	630.28 (575.99)	0.10
eGFR, ml/min/1.73 m^2^	67.87 (15.24)	63.72 (14.20)	<0.001
Smoking history, *n* (%)	11 (44)	—	—
Medication use			
Diabetes, *n* (%)	22 (88)	—	—
Hypertension, *n* (%)	14 (56)	—	—
Dyslipidemia, *n* (%)	19 (76)	—	—
Total teeth, *n*	20.84 (6.14)	—	—
Plaque index	1.02 (0.55)	—	—
Tongue coating index	2.08 (1.49)	—	—
PISA, mm^2^	380.41 (232.28)	—	—
IMT, mm	2.05 (0.74)	—	—

Values are presented as the mean (SD), unless otherwise indicated. Paired *t* tests were used to compare differences between week two and baseline. HbA1c, hemoglobin A1c; BMI, body mass index; BP, blood pressure; AST, aspartate aminotransferase; ALT, alanine aminotransferase; γ-GTP, *γ*-Glutamyl transpeptidase; HDL, high-density lipoprotein; LDL, low-density lipoprotein; hs-CRP, high-sensitivity C-reactive protein; eGFR, estimated glomerular filtration rate; PISA, periodontal inflamed surface area; IMT, carotid intima-media thickness.

**FIGURE 2:**
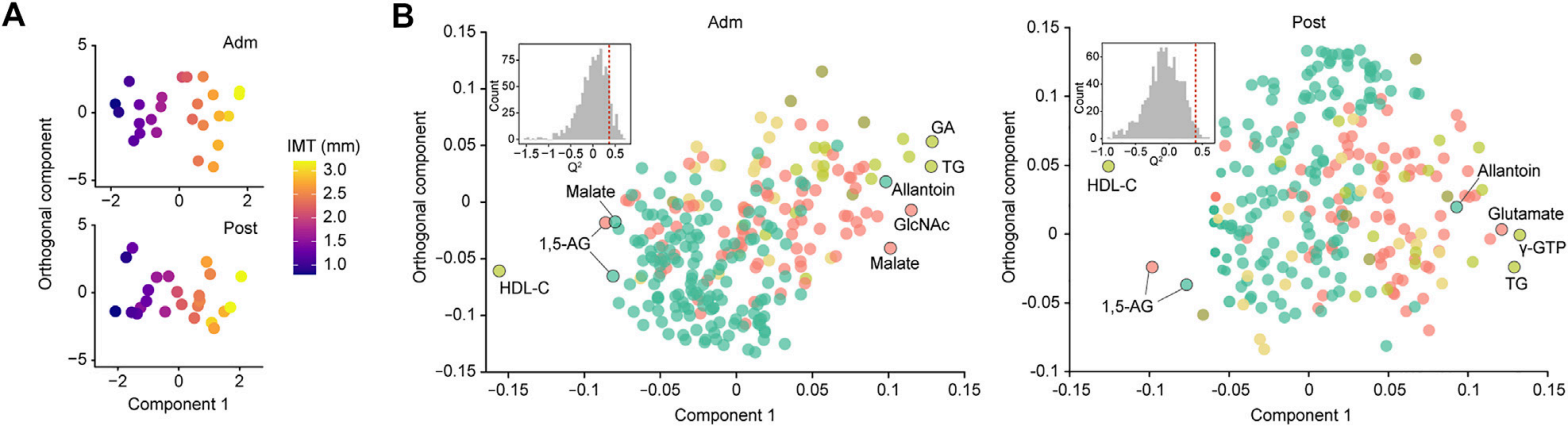
Global analysis of correlation of IMT with clinical and metabolomic features at admission (Adm) and after treatment (Post). **(A)** OPLS score plot showing distribution of study participants according to IMT. **(B)** OPLS loading plot showing color-coded distribution of predictors from different data blocks (pear: serum biochemical parameters, olive: urine biochemical parameters, light yellow: other clinical indices, red: plasma metabolome, mint: saliva metabolome), with the right side showing those associated with higher IMT. Inset shows statistical validation using permutation analysis (*n* = 999 permutations). GA, glycated albumin; TG, triglycerides; *γ*-GTP, *γ*-Glutamyl transpeptidase; HDL-C, high-density lipoprotein cholesterol; GlcNAc, *N*-acetylglucosamine; 1,5-AG, 1,5-anhydroglucitol.

**FIGURE 3:**
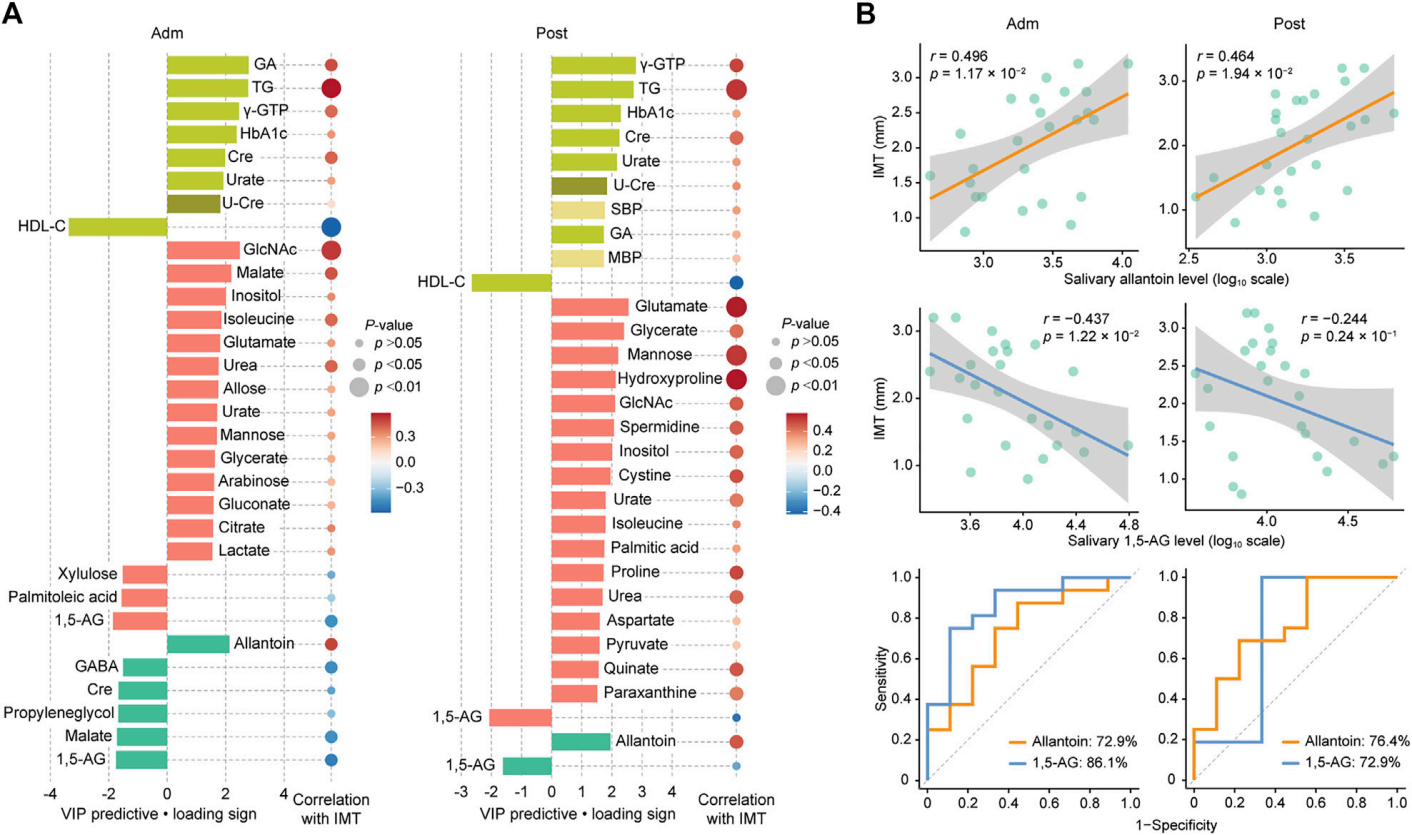
Top predictors for IMT from each data block at admission (Adm) and after treatment (Post). **(A)** Bar plots showing the variables with importance for projection of a predictive component (VIP predictive) higher than >1.5 (pear: serum biochemical parameters, olive: urine biochemical parameter, light yellow: other clinical indices, red: plasma metabolome, mint: saliva metabolome). Associations were also assessed using Spearman’s correlation. **(B)** Associations of IMT with salivary allantoin and 1,5-AG, and ROC curves for comparing discriminative performance for IMT >1.6 mm using salivary levels of allantoin and 1,5-AG. GA, glycated albumin; TG, triglycerides; γ-GTP, *γ*-Glutamyl transpeptidase; HbA1c, hemoglobin A1c; HDL-C, high-density lipoprotein cholesterol; Cre, creatinine; U-Cre, urine creatinine; SBP, systolic blood pressure; MBP, mean blood pressure; GlcNAc, *N*-acetylglucosamine; 1,5-AG, 1,5-anhydroglucitol; GABA, *γ*-Aminobutyric acid.

For more accurate characterization of the associations between IMT and clinical and metabolomic parameters at the baseline, and to examine the relative importance of variables in relation to IMT, OPLS was performed with IMT as a Y response variable (Figure 2A). The model showed a moderate predictive ability of 0.362 for *Q* ^2^ and reliable performance during the permutation test (*n* = 999 permutations; Figure 2B, inset). Top predictors for IMT from each data block [VIP predictive value > 1.5, p(corr) value <−0.3 or >0.3] included eight clinical markers, and 17 plasma and six salivary metabolites (Figure 3A). Notably, variables that best characterized IMT included the clinical markers HDL-C (*r* = −0.578, *p* = 0.002), glycated albumin (GA) (*r* = 0.472, *p* = 0.017), and TG (*r* = 0.581, *p* = 0.002), the plasma metabolites *N*-acetylglucosamine (GlcNAc) (*r* = 0.516, *p* = 0.008), malate (*r* = 0.461, *p* = 0.020) and inositol (*r* = 0.346, *p* = 0.091), and the salivary metabolites allantoin (*r* = 0.496, *p* = 0.012), 1,5-AG (*r* = −0.493, *p* = 0.012), and malate (*r* = −0.437, *p* = 0.029). Notably, salivary levels of allantoin and 1,5-AG showed AUC values of 0.729 and 0.861, respectively, for diagnosis in the high IMT group (>1.6 mm) (Figure 3B).

Two weeks of treatment during hospitalization resulted in significant improvements in multiple clinical parameters, including HbA1c, fasting plasma glucose, GA, and TG (Table 1), as demonstrated in our previous work (Taya et al., 2021). An OPLS model was then constructed using clinical and metabolomic parameters after 2 weeks of treatment for explanation of IMT variations. The model showed a moderate predictive ability of 0.398 for *Q* ^2^ and reliable performance during the permutation test (*n* = 999 permutations; Figure 2B, inset). Specifically, an unfavorable lipid profile remained predominant with the association between GA and IMT less pronounced, while amino acids became the predominant plasma metabolomic predictors for IMT. As for salivary metabolites, only allantoin and 1,5-AG remained relevant for IMT with a reduced predictive ability of 1,5-AG observed. Top predictors for IMT from each data block included the clinical markers γ-GTP (*r* = 0.503, *p* = 0.010), TG (*r* = 0.531, *p* = 0.0063), and HDL-C (*r* = −0.424, *p* = 0.035), the plasma metabolites glutamate (*r* = 0.571, *p* = 0.0029), glycerate (*r* = 0.425, *p* = 0.034), and mannose (*r* = 0.530, *p* = 0.0064), and the salivary metabolites allantoin (*r* = 0.464, *p* = 0.0194) and 1,5-AG (*r* = −0.244, *p* = 0.24) (Figure 3A). Additionally, salivary levels of allantoin and 1,5-AG showed AUC values of 0.764 and 0.729, respectively, for diagnosis in the high IMT group (Figure 3B).

**FIGURE 4:**
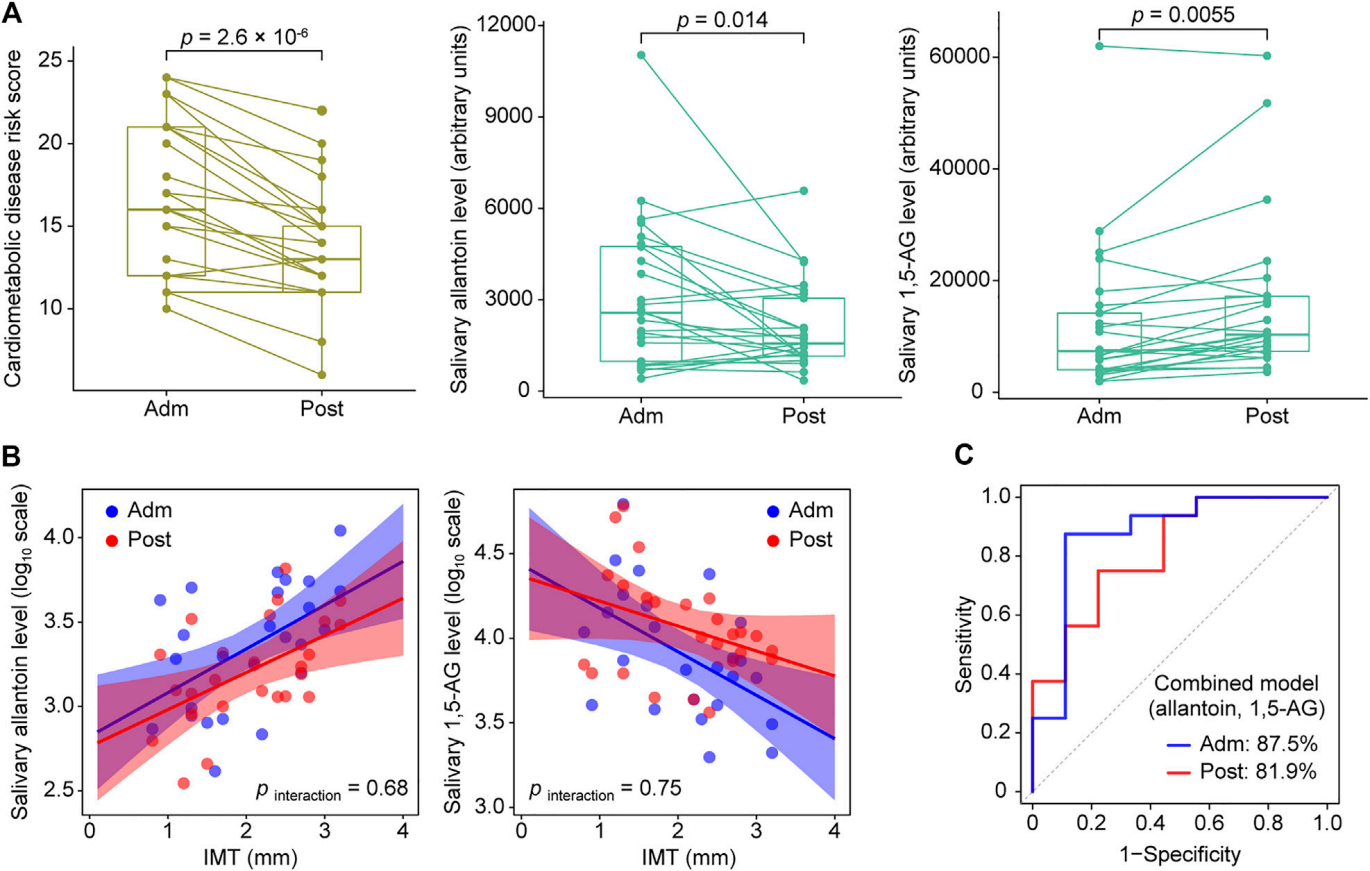
Responses of cardiometabolic risk factors and salivary metabolites following 2 weeks of intensive diabetes treatment. **(A)** Effects of intensive diabetes treatment on cardiometabolic disease risk score, and salivary levels of allantoin and 1,5-AG. The score for cardiometabolic disease risk was derived based on a composite score comprised of hemoglobin A1c, total cholesterol, triglycerides, high-density lipoprotein cholesterol, and high-sensitivity C-reactive protein values. *p* values shown are for within-group comparisons and were obtained with a paired *t* test for a comparison of the baseline score (Adm) with response after 2 weeks of treatment (Post). **(B)** Scatterplots of salivary markers with IMT according to hospitalized treatment. **(C)** ROC curves for comparing discriminative performance for IMT >1.6 mm using models combining salivary levels of allantoin and 1,5-AG at admission (Adm) as well as after treatment (Post).

Based on the OPLS results, we focused on salivary allantoin and 1,5-AG, and investigated the effects of hospitalized treatment on these salivary metabolites. A significant reduction in cardiometabolic disease risk score was noted following 2 weeks of hospitalization based on a composite score that summarized HbA1c, total cholesterol, TG, HDL-C, and hsCRP (*p* = 2.6 × 10^–6^) (Figure 4A). The two-week treatment also caused a significant decrease in the level of allantoin (*p* = 0.014) and an increase in 1,5-AG (*p* = 0.0055) in saliva (Figure 4A). However, that treatment did not modify the association between IMT and these metabolites (*p*_interaction_ > 0.05) (Figure 4B), while diagnostic accuracy for the high IMT group was satisfactory using models with combined salivary levels of these metabolites at the baseline (AUC = 0.875) as well as after treatment (AUC = 0.819) (Figure 4C). Collectively, while diabetes treatment in a hospitalization setting induced mitigation of cardiometabolic risk with accompanying alterations in salivary levels of allantoin and 1,5-AG, it did not have an effect modification on the relationship between these salivary metabolites and atherosclerotic burden in T2D patients, with the combination of salivary allantoin and 1,5-AG remaining highly discriminative for those at high risk for CVD regardless of glycemic control status.

## Discussion

The present findings demonstrated multivariate patterns of association of carotid wall thickening with metabolomic and clinical factors in patients with T2D. Additionally, they distinguished clinical and metabolomic parameters that change rapidly with improved glycemic control from those affected by relatively prolonged hyperglycemia, helping to find those more appropriate as markers of atherosclerosis, a cumulative disease. In particular, salivary allantoin and 1,5-AG remained the top metabolites reflecting IMT even after 2 weeks of inpatient glycemic control, revealing the potential utility of saliva testing for non-invasive assessment of carotid atherosclerosis severity, which might be useful for cardiovascular risk screening and monitoring of such patients.

Results of OPLS analysis with IMT as the outcome revealed clinical and metabolomic markers that exhibited a covariation with atherosclerotic burden in T2D patients. At the time of admission as well as after treatment, the severity of carotid atherosclerosis was associated with clinical markers in relation to an unfavorable lipid profile, such as lower HDL-C and higher TG, which is in agreement with several previous reports showing that increased TG/HDL-C ratio is an independent predictor of carotid atherosclerosis (Pacifico et al., 2014), as well as increased risk of CVD and all-cause mortality in patients with T2D (Wang et al., 2021b), and also non-diabetic subjects (Prasad et al., 2019; Sultani et al., 2020). The hsCRP is a known risk factor of CVD as well, but our analyses did not find it among its top predictors. The relatively large variance of hsCRP in our dataset may affect the results, however, we confirmed that exclusion of hsCRP did not change the main results in our OPLS models. Therefore, it is unlikely that the large variance of hsCRP undermines the validity of our models. The findings obtained in the present study also demonstrated GA to be more predictive of IMT than HbA1c at baseline. However, 2 weeks of intensive diabetes treatment attenuated the association of GA with IMT, which likely reflected improved glycemic control over a short term (Koga, 2014). It is considered that the associations between IMT and clinical markers shown in the present study corroborate the validity of the present measurements and metabolome analysis findings, as discussed following.

Regarding the relationship of plasma metabolites with IMT, GlcNAc was shown to have a stronger association with severity of carotid atherosclerosis at the baseline as compared to after treatment. Although there are no known clinical reports implicating an effect of plasma GlcNAc in development of atherosclerosis, a higher level of GlcNAc in plasma may well lead to increased protein modification *via* O-linked *β*-*N*-acetylglucosamine (O-GlcNAcylation), which has been implicated to be involved in development of diabetic cardiovascular complications (Chen et al., 2019; Chatham et al., 2020). Further investigation of the intricate relationship among plasma GlcNAc, O-GlcNAcylation, and atherosclerosis is warranted. The present results also indicated a number of known metabolites related to a higher atherosclerosis burden, such as inositol (Tzoulaki et al., 2019; Omori et al., 2020), isoleucine (Li et al., 2019), glutamate (Lehn-Stefan et al., 2021), urea (Omori et al., 2020), urate (Ishizaka et al., 2007), and hydroxyproline (Milanlouei et al., 2020). Additionally, data from OPLS analysis also indicated that 2 weeks of hospitalized treatment altered the profile of plasma metabolomic predictors for IMT, with amino acids predominant, which is likely associated with improved glycemic control status, though further validation is required.

A key finding in this study is that salivary allantoin was shown to be a potential indicator of IMT and cardiometabolic risk in patients with type 2 diabetes. Allantoin, produced by oxidation of urate, has been proposed as a biomarker for oxidative stress (Martinez-Moral and Kannan, 2019), while other studies have shown that urinary excretion of allantoin is correlated with atherosclerosis extension in mice (Li et al., 2015) and plasma allantoin is correlated with carotid atherosclerosis in humans (Santana et al., 2018). This is the first investigation to find an association between carotid atherosclerosis and salivary allantoin. However, allantoin was not annotated in the present plasma metabolomics results and, as noted in several other studies, no significant association was found between salivary allantoin and plasma urate, precluding mechanistic considerations. Nevertheless, it seems plausible that salivary excretion of allantoin becomes enhanced as a protective response to oxidative stress in association with diabetic angiopathy. Furthermore, since amelioration of hyperglycemia has been shown to reduce oxidative stress (Ohara et al., 2018), the lower level of oxidative stress induced by 2 weeks of intensive diabetes treatment is considered to have contributed to decreased salivary allantoin level, albeit the association with IMT was preserved. Although further research is needed, salivary allantoin might be a useful marker for reflecting the severity of atherosclerosis as part of monitoring CVD risk in patients with T2D.

Additionally, the present results demonstrated a correlation between IMT and 1,5-AG in both plasma and saliva samples, as well as a significant increase in salivary levels of 1,5-AG after 2 weeks of diabetes treatment. Previous studies have demonstrated that 1,5-AG can be used as a marker of short-term glycemic control and established it as a reliable T2D marker in addition to glucose (McGill et al., 2004). A positive association between plasma and salivary 1,5-AG has been reported by others (Mook-Kanamori et al., 2014; Jian et al., 2020) and was shown in our previous study (Sakanaka et al., 2021), which also presented findings demonstrating the diagnostic utility of salivary 1,5-AG for hyperglycemia when salivary 1,5-AG was used alone as well as in combination with salivary mannose. Furthermore, the results presented here showed that hospitalized treatment attenuated the association between 1,5-AG and IMT. Prior clinical studies did not find a significant association between plasma 1,5-AG and carotid atherosclerosis in examinations of subjects in the general population (Mukai et al., 2015), or of patients with T2D or hypertension (Karrei et al., 2004). Therefore, 1,5-AG is likely a marker of glycemic status rather than atherosclerosis. Nonetheless, it is important to note that the combination of salivary 1,5-AG with salivary allantoin may provide better prediction of cardiovascular risk in type 2 diabetes patients.

The current study has several limitations. Although the aim of this investigation was to comprehensively characterize biochemical and metabolic markers that exhibit covariation with the severity of carotid atherosclerosis by integrating multiple parameters obtained through detailed measurements, the small sample size may not allow for extrapolation of the findings. Future studies that replicate and further develop the present results using a larger sample are required. Additionally, we performed IMT measurement at the site of greatest thickness, including plaque lesions, according to guidelines used in Japan (Terminology and Diagnostic Criteria Committee and Japan Society of Ultrasonics in Medicine, 2009), which are different from European guidelines recommending that IMT measurement should be performed in a region free of plaque, and the distinction between IMT and plaque clearly made (Touboul et al., 2007). Nevertheless, several studies have shown that assessment of carotid plaque is more useful than IMT measured in plaque-free areas for predicting future CVD (Störk et al., 2004; Inaba et al., 2012). Additionally, it has been demonstrated that incorporation of carotid plaque in IMT measurements can better predict cardiovascular events as compared with information derived from plaque alone or IMT without inclusion of plaque (Baldassarre et al., 2012). Hence, the IMT definition employed in the present study seems suitable to achieve the goal of identifying metabolic signatures of atherosclerotic burden in diabetes patients.

## Conclusion

The present results show that a combination of salivary metabolites has robust associations with atherosclerotic burden in T2D patients and may be of high value for use in non-invasive identification of those at high risk for CVD in clinical practice. Additionally, they represent a new starting point for further investigations into the role of metabolites for exacerbation of diabetic macroangiopathy as well as their potential use for clinical diagnosis. It is considered that saliva testing will become even more widespread in the future, based on attention it has received due to the coronavirus pandemic, thus analysis of panels of metabolites in saliva may not only become an attractive alternative to blood tests for screening and monitoring of individuals with high risk for CVD, but might also help to reduce the daily burden faced by affected patients who must manage their symptoms over a long period of time. In addition, given the closer relationship developing between dentists and diabetologists, saliva testing during a regular dental visit may enable early warning of increased atherosclerotic burden in patients without subjective symptoms, thus strengthening the cooperation between medicine and dentistry for treatment of diabetes.

## Data Availability

The original contributions presented in the study are publicly available. These data can be found here: [https://www.metabolomicsworkbench.org/data/DRCCMetadata.php?Mode=Study&StudyID=ST001905] and [https://www.metabolomicsworkbench.org/data/DRCCMetadata.php?Mode=Study&StudyID=ST001906].

## Abbreviations

ALT, alanine aminotransferase; AST, aspartate aminotransferase; AUC, area under the curve; BMI, body mass index; BP, blood pressure; Cre, serum creatinine; CV-ANOVA, cross-validation analysis of variance; CVD, cardiovascular disease; FPG, fasting plasma glucose; GA, glycated albumin; GABA, *γ*-Aminobutyric acid; GC/MS, gas chromatography coupled with mass spectrometry; GlcNAc, *N-*acetylglucosamine; Gln, glutamine; eGFR, estimated glomerular filtration rate; γ-GTP, gamma-glutamyl transferase; HDL-C, high-density lipoprotein cholesterol; hsCRP, high-sensitivity C-reactive protein; IAA, indole acetic acid; LDL-C, low-density lipoprotein cholesterol; LOWESS, locally weighted scatter plot smoothing; MBP, mean blood pressure; OPLS, orthogonal partial least squares; PISA, periodontal inflamed surface area; QC, quality control; ROC, receiver operating characteristic; TG, triglycerides; U-Alb, urine albumin; U-Cre, urine creatinine; 1,5-AG, 1,5-anhydroglucitol.
